# Community evolution in retweet networks

**DOI:** 10.1371/journal.pone.0256175

**Published:** 2021-09-01

**Authors:** Bojan Evkoski, Igor Mozetič, Nikola Ljubešić, Petra Kralj Novak

**Affiliations:** 1 Department of Knowledge Technologies, Jozef Stefan Institute, Ljubljana, Slovenia; 2 Jozef Stefan International Postgraduate School, Ljubljana, Slovenia; Universite Lumiere Lyon 2, FRANCE

## Abstract

Communities in social networks often reflect close social ties between their members and their evolution through time. We propose an approach that tracks two aspects of community evolution in retweet networks: flow of the members in, out and between the communities, and their influence. We start with high resolution time windows, and then select several timepoints which exhibit large differences between the communities. For community detection, we propose a two-stage approach. In the first stage, we apply an enhanced Louvain algorithm, called Ensemble Louvain, to find stable communities. In the second stage, we form influence links between these communities, and identify linked super-communities. For the detected communities, we compute internal and external influence, and for individual users, the retweet h-index influence. We apply the proposed approach to three years of Twitter data of all Slovenian tweets. The analysis shows that the Slovenian tweetosphere is dominated by politics, that the left-leaning communities are larger, but that the right-leaning communities and users exhibit significantly higher impact. An interesting observation is that retweet networks change relatively gradually, despite such events as the emergence of the Covid-19 pandemic or the change of government.

## Introduction

With the ever-growing base of social media users, platforms such as Twitter are becoming a very valuable source of data for social analysis. Users on social media interact with each other, so it is natural to use graphs (where the users are nodes, and interaction between them are edges) to represent the structure of the user base. Nowadays, a lot of research in the field of complex networks is focused on social networks analysis. Due to the social media volatility, temporal analyses are needed for an in-depth understanding of the underlying phenomena. They can provide insights into the patterns and evolution of the social media landscape, and consequently to the society itself.

Change in the collective behaviour of groups in networks is referred to as community evolution [1], where communities in the networks are defined as groups of densely connected users. However, community detection methods are typically designed for static networks, and consequently have to be adapted for detecting changes in dynamic social media networks.

In our approach, we proceed by creating overlapping snapshots of the network through time, and detect communities in each snapshot. We then track evolution of relevant communities over time. Several developments are needed to detect community evolution in terms of the flow of members in, out and between the communities, as well as to track the changes in the community influence.

We illustrate our approach to community evolution on a set of Slovenian tweets during the last three years, roughly 13 million Twitter posts. Our initial research, where we performed a static community structure analysis of the data showed strong polarization of the detected communities along the political dimension. In the subsequent research, the basis of the current paper, we compared community structures between different manually selected time windows [2]. In the current paper, we describe a general set of techniques that enable semi-automatic analysis of the evolution of community structures and influence. These techniques make static community detection algorithms applicable to dynamic networks. We show a step-by-step application and insightful results of the proposed techniques on the Slovenian retweet networks.

### Related work

The temporal dimension is very valuable in modern analyses of complex networks. This has implications on how dynamic community discovery is designed and applied.

The related approaches mostly depend on the representation of time. One can group them into three types: static/edge-weighted, snapshots, and temporal networks [3]. The first community discovery methods were applied to the so-called “frozen in time” networks, where the temporal dimension is not explicitly represented. One operates with a single network (static or edge-weighted) that aggregates the whole period of interest. This absence of the time dimension has two historical reasons: the graph theoretic origin of the field, and the scarcity of data at the time when the field of complex networks emerged [4]. Aggregation strategies have severe limitations as they cannot capture dynamics, hence are not suitable for dynamic community detection. Consequently, the second representation emerged—temporally ordered series of network snapshots. This approach allows for efficient tracking of changes in the network structure, thus increasing the expressiveness of the models, but at a cost of higher analytical complexity [3]. Finally, temporal networks were proposed that allow for a complete and fine-grained description of the network dynamics [5]. The field of temporal network analysis is still under active development. Explicit temporal network representation is rarely used for dynamic community discovery, as it considerably increases the complexity of the models, and cannot easily make use of the existing community detection algorithms.

The snapshot approach crucially depends on the representation of time in the network relations. Two main scenarios were considered so far: perfect memory networks (also known as accumulative growth scenario), and limited memory networks. Perfect memory permits only aggregation of nodes and edges, where the old nodes/edges cannot disappear. The limited memory scenario allows for nodes/edges to disappear over time. This is suitable in social network analysis, where the edge disappearance could indicate the decay of social ties. The limited memory networks are implemented with various methods, including static, sliding, or dynamic-sized time windows, each method with its own strengths and weaknesses. In subsection Retweet networks we propose a combined method that circumvents the drawbacks of the existing strategies by building a weight-decaying sliding time window network. We then apply a snapshot selection method, described in subsection Selection of timepoints, where fixed static time windows that contain the most information about the dynamics of the communities are selected.

The second significant factor in dynamic community evolution is the way community detection is applied to the network snapshots [1, 3, 6–8]. Most of the existing approaches consider the following question: How do detected communities from one snapshot affect other snapshots (usually future-adjacent)? There are three groups of approaches: non-evolutionary, evolutionary, and coupling. The first one, also known as instant-optimal or two-stage approach, considers that communities already existing at time *t* depend only on the current state of the network at time *t*. A two-stage approach first detects communities at each snapshot, and then matches the detected communities [9, 10]. The obvious drawback of this approach is that the knowledge gained about the communities at snapshot *t*-1 is not used for communities at snapshot *t*. Yet, our method shows that this is not necessarily a weakness, when one is interested in detecting maximal changes in the community structure. In the evolutionary approach, also known as temporal trade-off, communities at snapshot *t* do not only depend on the network at the same time *t*, but also on the past evolution of the network [11–13]. The coupling approach shifts the focus from detecting communities at snapshot *t*, to community detection considering pairs of adjacent snapshots, or even the whole network evolution [14, 15].

Although there is a plethora of approaches, with all their advantages and drawbacks, most of the methods suffer from a common issue—the instability of community detection algorithms [16]. Community detection algorithms have different weaknesses, but the instability of the results is their common issue in the temporal scenarios. This is specially problematic in the evolutionary approach to dynamic community detection since the local instability also affects the time dependent communities. In other words, a “bad” run of the community detection algorithm influences the results of detection at the subsequent snapshots. This instability is also an issue for the community evolution analysis in our work, as one cannot distinguish if the community differences are due to the real-world events reflected in the dynamic complex network, or are they simply a consequence of the instability of the algorithm. To address this issue, we propose an Ensemble Louvain algorithm which to some extend solves the instability of the well-known Louvain algorithm for community detection.

### Structure of the paper

The main body of the paper is in the Results section. We start with a brief overview of the data collected in the Structure of the Twitter data subsection. In the Retweet networks subsection we describe how the network snapshots are created. Network partitions, generated by an extension of the Louvain algorithm, are described in the Community detection subsection. Evolving communities in adjacent partitions are compared in the Structure of the Measuring community similarity subsection. In Selection of timepoints we show how to select just a few relevant timepoints out of the whole timeline sequence. Two types of transitions are depicted in the Visualization of community transitions subsection. We then define internal and external influence in the Structure of the Identification of super-communities subsection. In the last subsection Retweet h-index influence we show the most influential users in our dataset. In Conclusions we wrap up our approach to community evolution and present main plans for future research. The Methods section provides some additional details. The Data collection subsection describes a specialized tool used for Twitter acquisition. Ensemble Louvain gives details of the community detection algorithm applied, and some preliminary evaluation results. The last subsection on BCubed measure of community similarity defines the measures used throughout the paper.

## Results

### Twitter data

Social media, and Twitter in particular, are widely used to study various social phenomena. For this study, we collected a set of all Slovenian tweets in the three year period, from January 1, 2018 until December 28, 2020. The set of almost 13 million tweets represents an exhaustive collection of Twitter activities in Slovenia. See the Methods section for details of the Twitter data acquisition.

**Fig 1:**
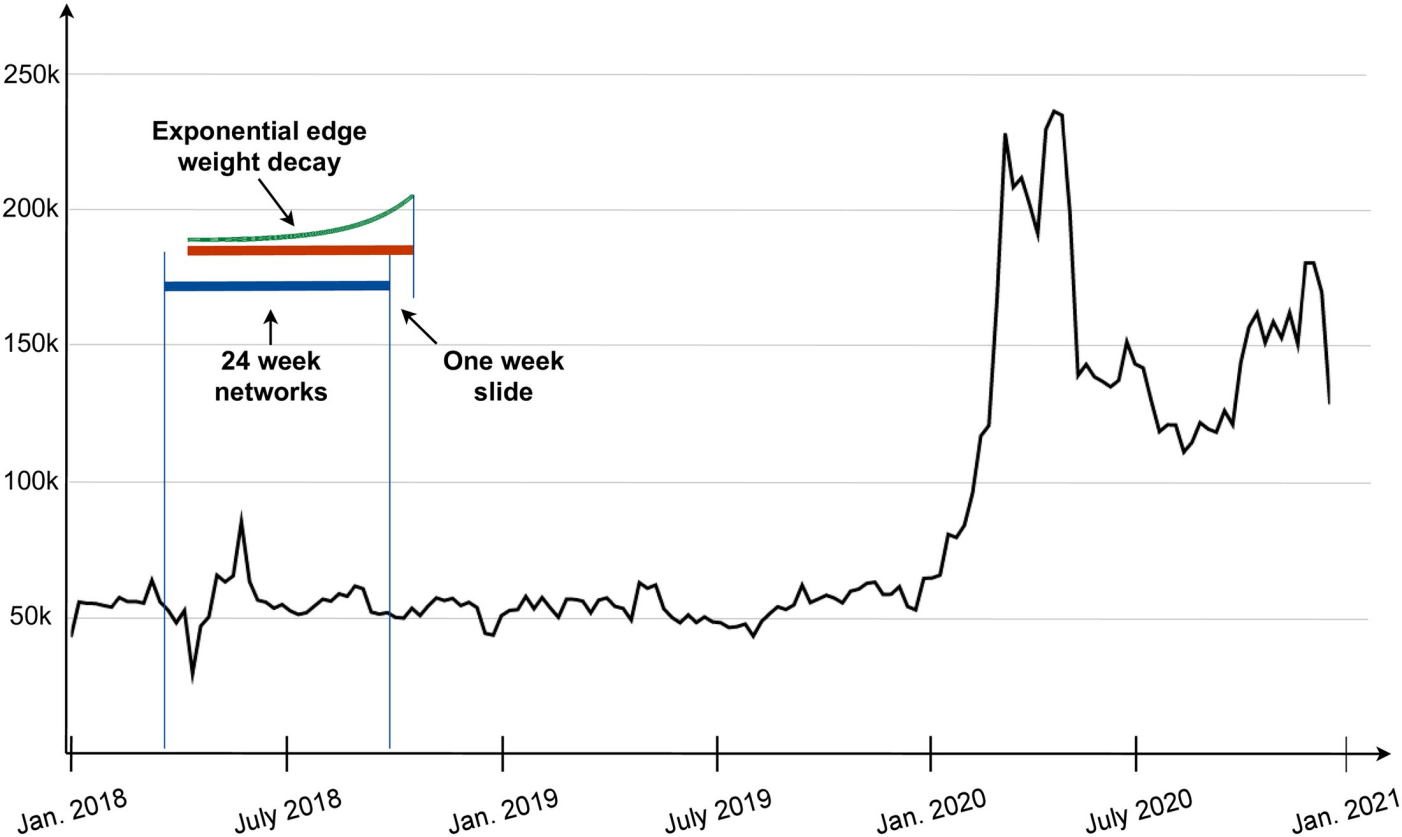
Weekly volume of Slovenian Twitter data, collected over the three year period. The retweet network observation window is 24 weeks (blue and red lines), with exponential weight decay (half-time of 4 weeks, green curve), and one week sliding window (difference between the red and blue line). Note a large increase of Twitter activities at the emergence of the Covid-19 pandemic, which also coincided with the change of the left-wing to the right-wing government in Slovenia.

Fig 1 shows the weekly volume of tweets collected during the three years. The number of tweets is fairly stable, around 50,000 per week, until the emergence of the Covid-19 pandemic in March 2020. At this point, we observe a four-fold increase of Twitter activities. This also coincides with a change of government in Slovenia, from the left-wing to the right-wing. A minor peak can also be observed around June 2018, at the time of the snap parliamentary elections. It turns out that most of the tweets are related to politics, and, after March 2020, to policies concerning the handling of the pandemic. The following is a list of the most important political events in Slovenia during the last three years:

- March 14, 2018—left-wing government resignation ($PM-sep14-sep18),
- June 8, 2018—snap parliamentary elections,
- September 13, 2018—new left-wing government formation ($PM-sep18-mar20),
- January 27, 2020—left-wing government resignation ($PM-sep18-mar20),
- March 13, 2020—right-wing government formation ($PM-mar20-now),
- March, 2020—emergence of the Covid-19 pandemic in Slovenia.

In parenthesis we give anonymized Twitter handles of the Slovenian prime ministers (PM) at the time since they have important roles in their respective communities. PLoS ONE policy requires to remove information which identifies and names individual Twitter users.

### Retweet networks

Twitter provides different forms of interactions between the users: follows, mentions, replies, and retweets. The most useful indicator of social ties between the Twitter users are retweets. When a user retweets a post, it is distributed to all of its followers, just as if it were an originally authored post. Users retweet content that they find interesting or agreeable. Despite the fact that it does not always signify an endorsement (e.g., tweets by the former U.S. president Trump), in large number of cases retweets indicate links between the like-minded users. In particular, in politics retweets very well reflect the actual political alignments and influence. For example, it was demonstrated that political parties and nationalities of the members of the European Parliament can be reconstructed solely from their retweet activities [17]. There is also a correspondence between the co-voting and retweeting in the European Parliament, while higher Twitter activity was observed for the right-wing parties [18]. In the case of Brexit, the Leave proponents showed much higher activity and influence on Twitter than the Remain proponents [19].

A retweet network is a directed graph *G*. The nodes are Twitter users and the edges are retweet links between the users. An edge is directed from the user *A* who posts a tweet to the user *B* who retweets it. The edge weight is the number of retweets posted by *A* and retweeted by *B*. For the whole three year period of Slovenian tweets, there are in total 18,821 users (nodes) and 4,597,865 retweets (sum of all weighted edges).

To study dynamics of the retweet networks, we form several network snapshots from our Twitter data. In particular, we select a network observation window of 24 weeks (about six months), with a sliding window of one week. This provides a relatively high temporal resolution between subsequent networks, but later we show how to select the most relevant intermediate timepoints (see subsection Selection of timepoints). Additionally, we employ an exponential edge weight decay, with half-time of 4 weeks (see Fig 1). The reason for this temporal weight decay is to eliminate the effects of the trailing end of the moving network snapshots.

The set of network snapshots thus consists of 133 overlapping observation windows, with temporal delay of one week. The snapshots start with network *G*_0_ (January 1, 2018–June 18, 2018) and end with network *G*_132_ (July 13, 2020–December 28, 2020).

### Community detection

Informally, a network community is a subset of nodes more densely linked between themselves than with the nodes outside the community. There are several formal definitions of communities and different methods to detect them. A practical review that provides strengths and weaknesses of the most popular methods is provided in [20].

A standard community detection method is the Louvain algorithm [21]. Louvain finds a partitioning of the network into communities, such that the modularity of the partition is maximized. For a partition, the modularity measures the density and structure of its communities: the fraction of edges within the communities, as compared to the expected fraction of randomly distributed edges in the network [22]. The Louvain algorithm is computationally efficient, well suited for large networks, and does not require ex-ante assumptions about the number or size of the communities [23].

However, there are several problems with the modularity maximization [20]. One, from a theoretical point of view, is that there are typically exponentially many distinct partitions whose modularity scores are very close to the global maximum [24]. As a consequence, from a practical point of view, the Louvain algorithm yields different partitions for different trials on the same network (see Fig 7 in Methods for an example).

We address this instability problem of Louvain by applying the Ensemble Louvain algorithm. We run 100 trials of Louvain and compose communities with nodes that co-occur in the same community above a given threshold, 90% of the trials in our case. This results in relatively stable communities of approximately the same size as produced by individual Louvain trials. Details of the Ensemble Louvain algorithm are in the Methods section.

Our 133 retweet network snapshots are directed graphs, *G*_0_, …, *G*_132_, with weighted edges. For community detection, we transform them into undirected graphs. When a pair of nodes is linked with two weighted edges of the opposite direction, we create an undirected edge with the sum of the original edge weights. When a pair of nodes is linked with a single directed edge, we simply drop the direction. We then run the Ensemble Louvain on all the 133 undirected network snapshots, resulting in 133 network partitions, *P*_0_, …, *P*_132_.

### Measuring community similarity

The sequence of network partitions, *P*_0_, …, *P*_132_, produced by Ensemble Louvain, varies. Community structure changes, new nodes join some communities, and some nodes disappear from a network snapshot. To study community evolution, one has to compare subsequent network partitions.

There are several measures to evaluate network communities, in particular in relation to the “ground truth”. Two widely used measures are Adjusted Rand Index (ARI) [25] and Normalized Mutual Information (NMI) [26]. In this study we use the BCubed measure, extensively evaluated in the context of clustering [27]. BCubed yields evaluation results similar to ARI and NMI (see Fig 7 in Methods). However, there are several advantages of BCubed, useful in the context of community evolution. In particular, we extend the BCubed measure to account for the new and lost nodes between two network partitions.

BCubed decomposes evaluation into calculation of precision and recall of each node in the network. The precision (*Pre*) and recall (*Rec*) are then combined into the *F*_1_ score, the harmonic mean:

$$\begin{array}{c} F_{1}=2\;\frac{Pre\cdot Rec}{Pre+Rec}. \end{array}$$

Details of computing *Pre* and *Rec* for individual nodes, communities and network partitions are in the Methods section. Here we just emphasize that our extended BCubed *F*_1_ is different and more general than the *F*_1_ score proposed by Rossetti [28].

In the following, we refer to our extended BCubed *F*_1_ score as simply *F*_1_. When we compare two network partitions, *P*_*t*_ and *P*_*t*−1_, we consider a partition earlier in time *P*_*t*−1_ as “ground truth”, and evaluate the subsequent partition *P*_*t*_ with respect to the previous one. We write *F*_1_(*P*_*t*_|*P*_*t*−1_) to denote the similarity of *P*_*t*_ to *P*_*t*−1_. *F*_1_ ranges from 0 to 1, where increasing *F*_1_ indicates higher similarity between the two partitions.

There are two special cases of *F*_1_. When the two partitions consist of the same nodes, just distributed differently between the communities, *F*_1_ degenerates into *core-F*_1_. *core-F*_1_ is directly compatible to ARI and NMI. When the two partitions differ in the constituent nodes, i.e., there are new and lost nodes, one can compute the theoretical maximum similarity, *max-F*_1_, where all the nodes common to both partitions (the intersection) are assumed to be in one community. *max-F*_1_ thus measures similarity of two sets and is directly related to the Jaccard index. See Methods for details.

**Fig 2:**
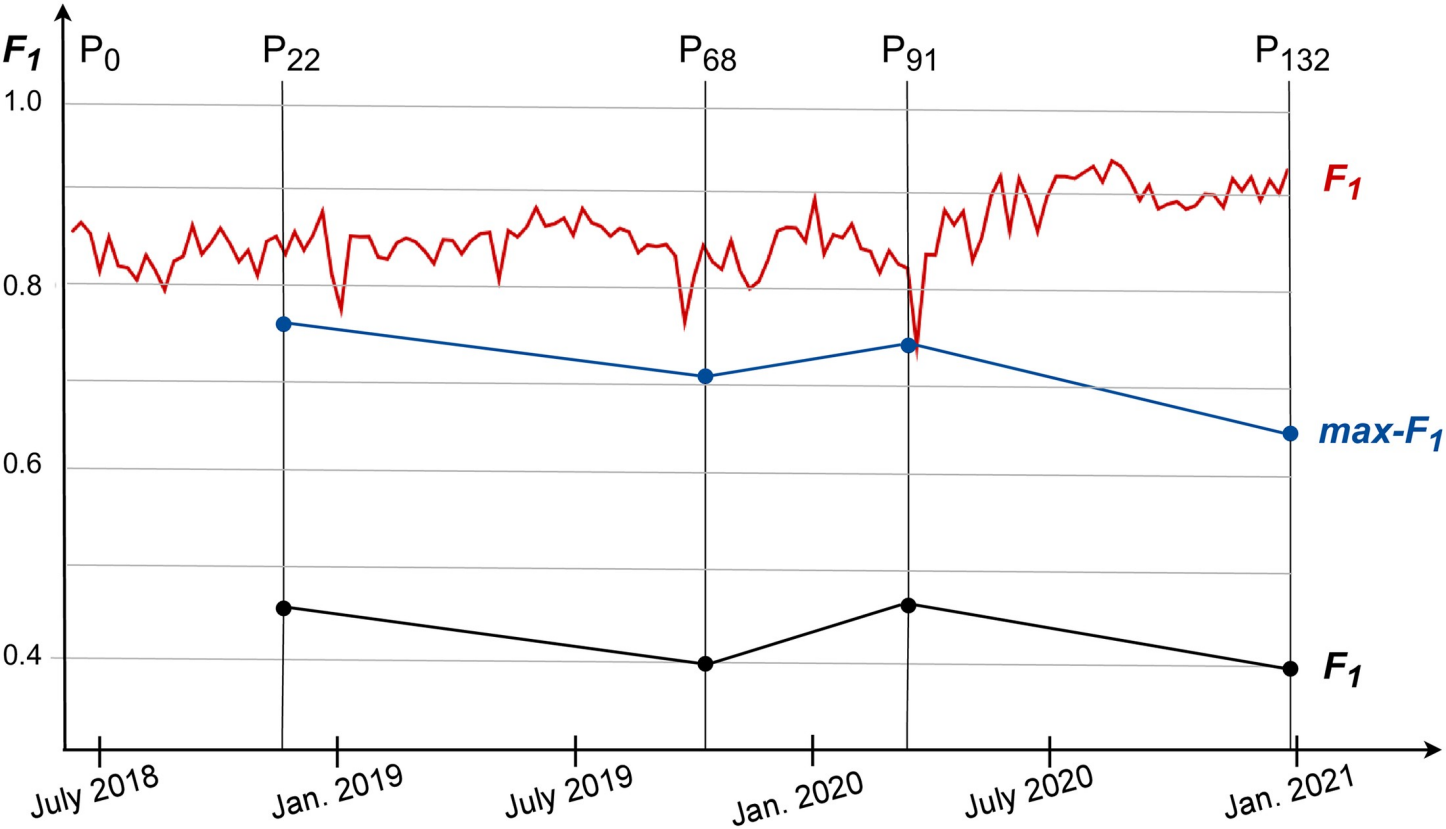
Differences between the adjacent network partitions measured by the *F*_1_ score. The red line at the top shows weekly differences *F*_1_(*P*_*t*_|*P*_*t*−1_) at timepoints *t* = 1, 2, …, 132. The five selected partitions are denoted by *P*_0_, *P*_22_, …, *P*_132_. The middle blue line shows the theoretical maximum *max-F*_1_ differences between distant partitions at the selected timepoints *t* = 0, 22, 68, 91, 132. The bottom black line shows the standard *F*_1_ differences.

Fig 2 (red line) shows pairwise *F*_1_(*P*_*t*_|*P*_*t*−1_) differences between the retweet network partitions at weekly timepoints *t* = 1, 2, …, 132. The *F*_1_ scores are relatively high, typically in the range [0.8, 0.9]. The largest negative peak, indicating the highest dissimilarity, *F*_1_(*P*_92_|*P*_91_) = 0.74, occurs between the partitions which end on March 16 and 23, 2020, respectively. These dates closely follow the change of government in Slovenia and first policy reactions to the emergence of the Covid-19 pandemic.

### Selection of timepoints

The weekly differences between the network partitions are relatively low. The retweet network communities apparently do not change drastically at this relatively high time resolution. Moving to lower time resolution means choosing timepoints which are further apart, and where the network communities exhibit more pronounced differences.

We formulate the timepoint selection task as follows. Let assume that the initial and final timepoints are fixed, corresponding to the partitions *P*_0_ and *P*_*n*_, respectively. For a given *k*, select *k* intermediate timepoints such that the differences between the corresponding partitions are maximized, i.e., the *F*_1_ scores are minimized:

$$\begin{array}{c} min\;(\sum_{i=1}^{k}F_{1}\left(P_{i}|P_{i-1}\right)+F_{1}\left(P_{n}|P_{k}\right)). \end{array}$$

There are (n−1k)·k! possible selections of timepoints, i.e., the number of selections grows exponentially with *k*. We therefore propose a simple heuristic algorithm which finds *k* approximate timepoints. The algorithm works top-down and starts with the full, high resolution timeline with *n* + 1 timepoints, *t* = 0, 1, …, *n* and corresponding partitions *P*_*t*_. At each step, it finds a triplet of adjacent partitions *P*_*t*−1_, *P*_*t*_, *P*_*t*+1_ with minimal differences:

$$\begin{array}{c} max\;(F_{1}\left(P_{t}|P_{t-1}\right)+F_{1}\left(P_{t+1}|P_{t}\right)) \end{array}$$

and eliminates *P*_*t*_ from the timeline. At the next step, the difference *F*_1_(*P*_*t*+1_|*P*_*t*−1_) fills the gap of the eliminated timepoint *P*_*t*_. The algorithm thus finds the *k* (non-optimal) timepoints in *n* − 1 − *k* steps. While efficient, this approach to the relevant timepoint selection is not suitable for incremental, stream-based network processing since it assumes that the final timepoint is fixed.

For our retweet networks, we experimented with several values of *k* and eventually settled with *k* = 3 which provides much lower, but still meaningful time resolution. This resulted in the selection of the following network partitions: *P*_0_, *P*_22_, *P*_68_, *P*_91_, *P*_132_. Fig 2 shows the *F*_1_ differences (black line) between the adjacent partitions.

The selected timepoints are on average 26 weeks apart, varying between five and ten months. The differences between the network partitions are increasing with temporal distance, but are still relatively uniform, *F*_1_ is in the range [0.4, 0.5]. Due to these small differences, the timepoint selection procedure is not very robust. The selected timepoints should be considered approximate and can vary for several weeks in both directions. As a consequence, the selected timepoints should not be interpreted as indicators of specific events at specific dates, but should rather help in understanding longer terms qualitative transitions in community evolution.

Fig 2 also shows the theoretical maximum differences *max-F*_1_ (blue line), where it is assumed that all the common nodes in two adjacent partitions are in one community, and only the intersection size and the number of new and lost nodes affect the score. The *max-F*_1_ scores, dropping from 0.77 to 0.63, show increasing fluctuation of nodes in and out of the partitions. In the next subsection we show two visualizations of transitions between these five network partitions.

### Visualization of community transitions

We present two visualizations of transitions between selected network partitions as Sankey diagrams. A Sankey diagram is a type of flow diagram in which the width of the bands is proportional to the flow rate.

**Fig 3:**
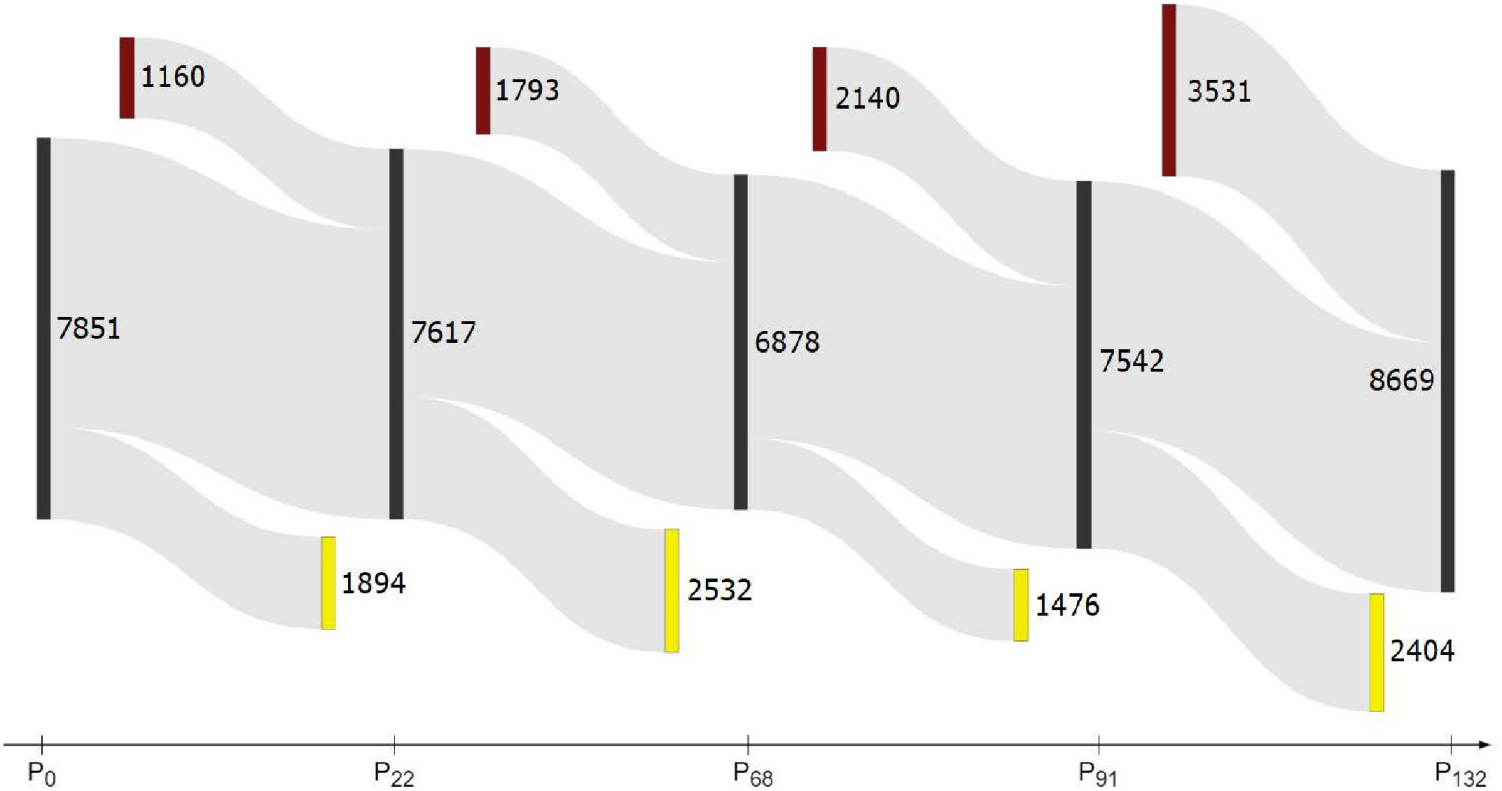
A Sankey diagram showing major transitions between the five selected timepoints *P*_0_, *P*_22_, …, *P*_132_. The numbers indicate core nodes (black), new nodes (brown, at top), and lost nodes (yellow, at bottom) between two adjacent network partitions. The differences between the adjacent partitions are quantified by *max-F*_1_(*P*_*i*_|*P*_*i*−1_), shown with blue line in Fig 2. Note a relatively large in- and out-flow of new and lost nodes between the partitions.

In Fig 3 we show inflows of new nodes, outflows of lost nodes, and transition flows of core (intersection) nodes between the selected network partitions. Note that only about half of the nodes remain in the core transitions. Therefore it is crucial that the community similarity measure takes new and lost nodes into account. This diagram ignores the internal community structure, and corresponds to the theoretical maximum *max-F*_1_ (shown in Fig 2) where all the core nodes are assumed to be in the same community.

**Fig 4:**
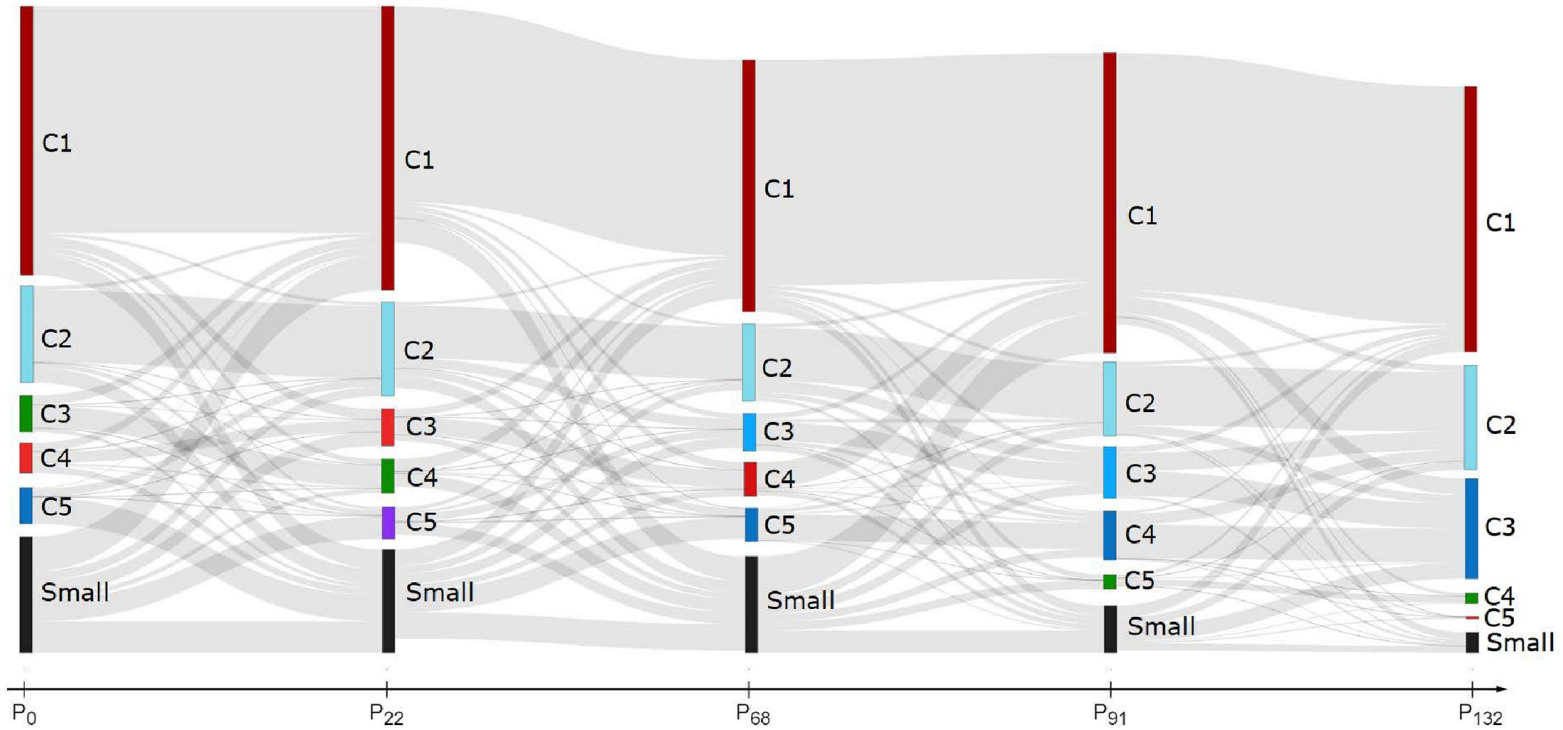
A Sankey diagram showing transitions between the five largest communities C1, …, C5 at the selected timepoints *P*_0_, *P*_22_, …, *P*_132_. The remaining smaller communities are labeled as Small, and new and lost nodes are not shown here. The differences between the adjacent partitions are quantified by *F*_1_(*P*_*i*_|*P*_*i*−1_), shown with black line in Fig 2. The left-leaning communities are in shades of red, the right-leaning communities are in shades of blue, and the Sports community is green.

Fig 4 is more detailed and shows the internal community structure of the cores, with the top five communities C1, …, C5 at each selected timepoint. All the remaining smaller communities are appended together into a single Small community.

The top communities were manually scanned for the most influential users (see subsection Retweet h-index influence) and discussion topics. It turns out that most of the communities are structured around political figures (either politicians, public figures, or journalists with clear political orientation) [29], and that political and ideological topics are prevailing [30]. Thus, the top communities can be classified into three categories: left-leaning (most influential users are part of the left-wing structures), right-leaning (most influential users are part of the right-wing), and Sports (users and topics are clearly related to sports). In Fig 4, the left-leaning communities are in shades of red and the right-leaning communities are in shades of blue. The only non-political community is Sports, in green, represented by the following sequence of communities:

$$\begin{array}{c} C3_{0}\mapsto C4_{22}\mapsto {\left(C\subset Small\right)}_{68}\mapsto C5_{91}\mapsto C4_{132}. \end{array}$$

A community *Ci* at timepoints *t* is denoted by *Ci*_*t*_. Note that at timepoint *t* = 68 the Sports community is absorbed into the Small community.

The political communities are considerably larger than Sports. Let us first consider some right-leaning communities, which feature the current Slovenian prime minister of the right-wing government, with an anonymized Twitter handle $PM-mar20-now. He was initially a member of relatively small communities that at timepoint *t* = 22 did not even make it into the top five:

$$\begin{array}{c} C5_{0}\mapsto {\left(C\subset Small\right)}_{22}\mapsto C5_{68}\mapsto C4_{91}\mapsto C3_{132}. \end{array}$$

Only after the right-wing government took over in March 2020 (timepoints *t* = 91, 132) did his community grow considerably.

On the political left-wing, there is the *C*1 community that grows and shrinks with time, but remains by far the largest community throughout the three year period. The former Slovenian prime minister (between September 2018 and March 2020), with an anonymized Twitter handle $PM-sep18-mar20, was a member of *C*1 for most of the time:

$$\begin{array}{c} C1_{0}\mapsto C1_{22}\mapsto C4_{68}\mapsto C1_{91}\mapsto C1_{132}. \end{array}$$

Only in the second half of his government (*t* = 68) did he feature prominently in his own community *C*4. The left-wing Slovenian prime minister before him (until March 2018), with an anonymized Twitter handle $PM-sep14-sep18, was initially a member of smaller communities on the left-wing, and recently joined *C*1:

$$\begin{array}{c} C4_{0}\mapsto C3_{22}\mapsto C4_{68}\mapsto C1_{91}\mapsto C1_{132}. \end{array}$$

It is interesting to observe the official Slovenian government Twitter account @vladaRS. It moves from the left-leaning to the right-leaning communities as the left-wing government is replaced by the right-wing one, but with some delay:

$$\begin{array}{c} C4_{0}\mapsto C3_{22}\mapsto C4_{68}\mapsto C1_{91}\mapsto C3_{132}. \end{array}$$

@vladaRS matches the $PM-sep14-sep18 community at *t* = 0, 22, the $PM-sep18-mar20 community at *t* = 68, 91, and the $PM-mar20-now community at *t* = 132. This is another piece of evidence that retweet communities evolve gradually and that it takes a while before events with a high impact are reflected in a new community structure.

To further characterize political polarization and community evolution, we now turn attention from the community membership to the retweet links between the communities.

### Identification of super-communities

Twitter users differ in how prolific they are in posting tweets, and in the impact these tweets make on the other users. One way to estimate the influence of a Twitter user is to consider how often are its posts retweeted. Similarly, the influence of a community can be estimated by the total number of retweets of their posts. Retweets within the community indicate internal influence, and retweets outside of the community indicate external influence. This approach to characterize influential users and communities was already applied to a wide range of environmental issues discussed on Twitter [31].

In this subsection we focus on community influence and subsequent identification of super-communities. Another measure of individual influence is described in the next subsection Retweet h-index influence. In our retweet networks, the number of retweets is represented by the weighted out-degree of a node. Let *W*_*ij*_ denote the sum of all weighted edges between communities *C*_*i*_ and *C*_*j*_. The average community influence *I* is defined as:

$$\begin{array}{c} I\left(C_{i}\right)=\frac{\sum_{j}W_{ij}}{|C_{i}|}, \end{array}$$

i.e., the weighted out-degree of *C*_*i*_, normalized by its size. The influence *I* consists of the internal *I*_*int*_ and external *I*_*ext*_ component, *I* = *I*_*int*_ + *I*_*ext*_, where

$$\begin{array}{c} I_{int}\left(C_{i}\right)=\frac{W_{ii}}{|C_{i}|}, \end{array}$$

and

$$\begin{array}{c} I_{ext}\left(C_{i},C_{j}\right)=\frac{\sum_{i\neq j}W_{ij}}{|C_{i}|}. \end{array}$$

**Fig 5:**
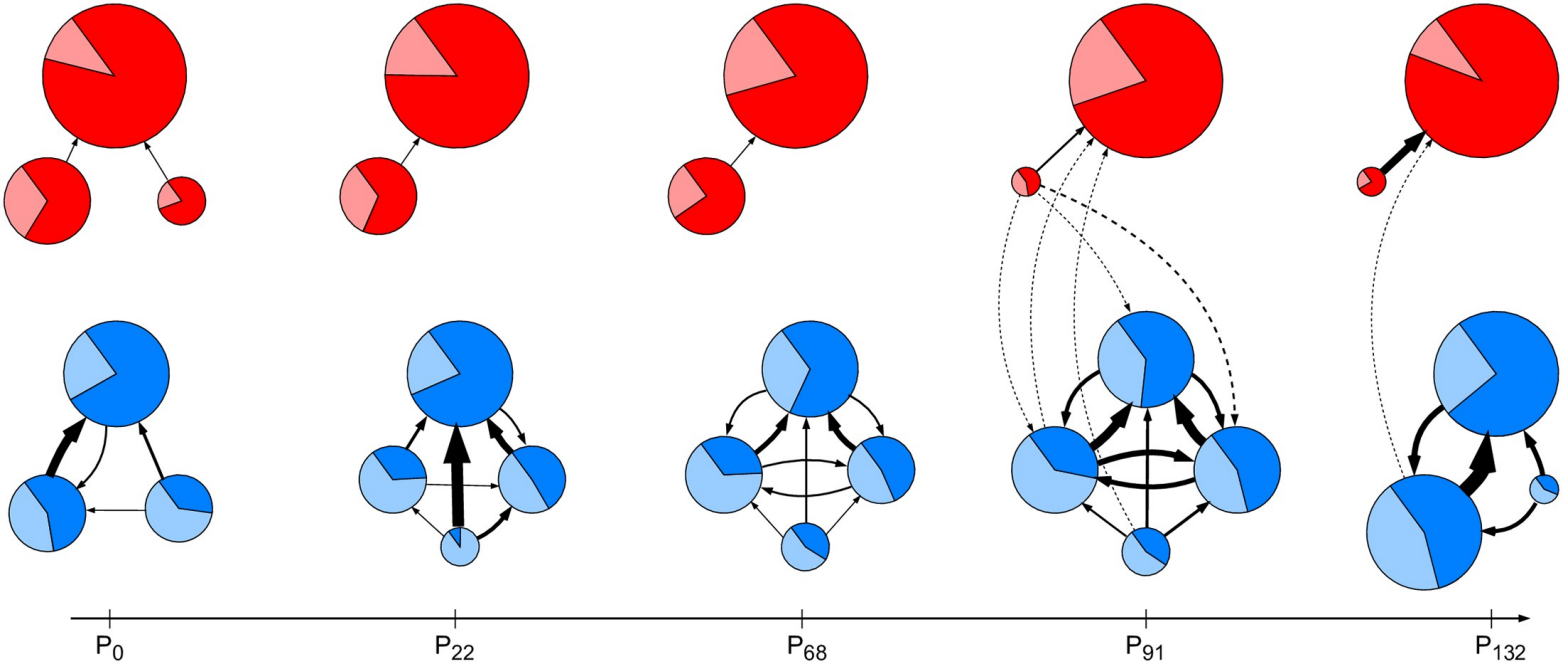
Identification of super-communities from the meta-networks. Nodes are detected communities C1, …, C7 at different timepoints, and edges denote average external influences. A node diameter is proportional (cube-root) to the number of community members, darker area corresponds to the internal influence, and lighter area to the external influence. Red communities are part of the Left-wing super-community, blue communities are part of the Right-wing super-community, and the remaining Sports community is not shown. Dashed edges show rare and relatively weak links between the Left- and Right-wing communities.

We compute internal and external influence of the retweet communities detected at the selected timepoints *t* = 0, 22, 68, 91, 132. The communities which are politically left- or right-leaning are shown in Fig 5, the Sports community is omitted. A community, proportional to its size, is depicted as a pie chart, indicating its internal and external influence. A pair of communities *C*_*i*_, *C*_*j*_ is linked by a weighted directed edge from *C*_*i*_ to *C*_*j*_, with the weight equal to the external influence *I*_*ext*_(*C*_*i*_, *C*_*j*_).

The meta-networks in Fig 5 support clear identification of two super-communities: Left-wing and Right-wing. A super-community exhibits relatively strong external influence links between its constituent communities. However, there are considerable differences between the Left-wing and Right-wing super-communities. The Left-wing is larger, and its communities have higher internal influences. The Right-wing, on the other hand, has stronger inter-community links, its communities have higher external influences, and appears more cohesive. Note that there are barely any links between the Left-wing and Right-wing communities, a characteristics of echo chambers and political polarization [32].

**Fig 6:**
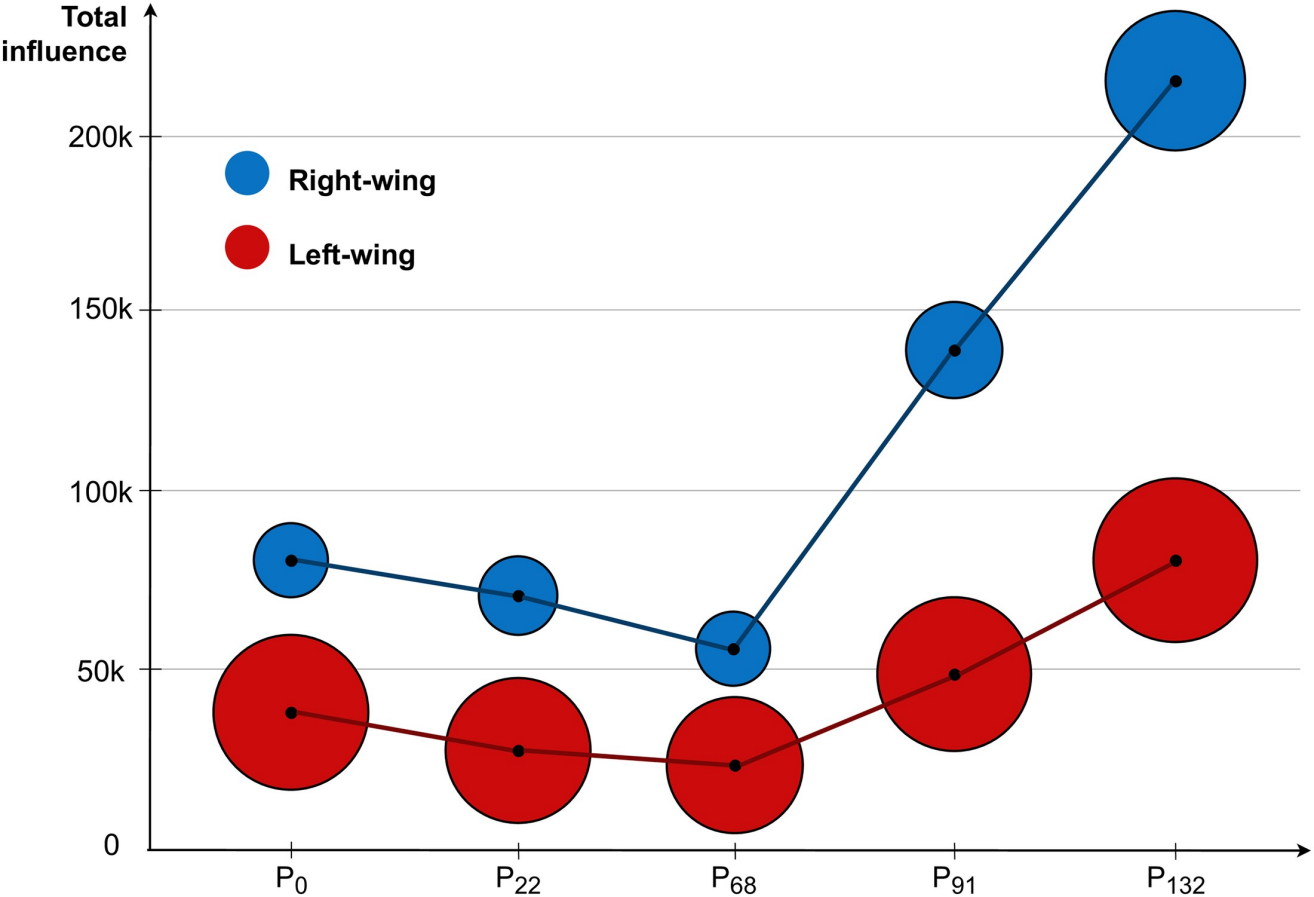
Total weighted out-degree influence for both super-communities. A super-community size is proportional to the number of its members. Total influence is the sum of weighted out-degree influences of all super-community members. Note that the influence of the Right-wing super-community is at least twice as large as the influence of the Left-wing super-community and increasing with time, despite the fact that it is considerably smaller.

In Fig 6 we show the total influence of both super-communities. Total influence of a super-community is the sum of weighted out-degrees of all its members, without normalization. The Right-wing super-community is typically half the size of the Left-wing, approaching in size only at the last timepoint *t* = 132. However, the influence of the Right-wing is always considerably higher, with the gap even increasing after the right-wing government took over in March 2020 (timepoints *t* = 91, 132).

### Retweet h-index influence

Weighted out-degree is a useful measure of influence for communities and super-communities. However, we propose a different measure of influence for individual Twitter users. The user influence is estimated by their retweet h-index, an adaptation of the well known Hirsch index [33] to Twitter. The retweet h-index takes into account the number of tweets posted, as well as the impact of individual posts in terms of retweets.

A user with an index of *h* has posted *h* tweets and each of them was retweeted at least *h* times. Let *RT* be the function that returns the number of retweets for each original post. The values of *RT* are ordered in decreasing order, from the largest to the lowest, and *i* indicates the ranking position in the ordered list. The h-index is then computed as follows:

$$\begin{array}{c} \text{h-index}\left(RT\right)=\underset{i}{\text{max}}\;\text{min}\left(RT\left(i\right),i\right). \end{array}$$

To the best of our knowledge, the retweet h-index was first used on Twitter data in the context of Brexit, to measure the influence of the Leave and Remain proponents [19]. Later, this measure of influence was termed a retweet h-index [34], a term we also adopt here.

We compute the h-index and the h-index rank for all the users on Slovenian Twitter during the three year period. For each super-community, Left-wing and Right-wing, we show the top ten most influential users by h-index, ordered by the h-index rank (Table 1). The users are ranked for the overall three year period, but the h-index and relative ranks are also provided for the selected timepoints *t* = 0, 22, 68, 91, 132. Two of the top Twitter users, @vladaRS and @ukclj, do not remain in the same super-community, but move from the Left-wing to the Right-wing as the government changed.

**Table 1:** Top ten influential users from each super-community, ranked by the overall h-index. Individual Twitter users are anonymized and their handles start with $. Left-to-Right denotes users which moved from the Left-wing to the Right-wing super-community (@vladaRS: the official Slovenian government account, and @ukclj: University Medical Centre Ljubljana). The top users in each super-community are $PM-mar20-now (current prime minister), and $PM-sep18-mar20 (former prime minister), respectively. Each user is assigned the h-index rank (h-rank), the h-index (h-ind) for the overall three year period and the five selected timepoints (*P*_0_, …, *P*_132_), and the overall unweighted out-degree (out-deg). Note that the top Left-wing influential users barely reach the h-index rank of top 100.

User	Overall			*P*_0_		*P*_22_		*P*_68_		*P*_91_		*P*_132_	
	h-rank	h-ind	out-deg	h-rank	h-ind	h-rank	h-ind	h-rank	h-ind	h-rank	h-ind	h-rank	h-ind
**Right-wing**:													
$PM-mar20-now	1	168	2621	1	93	1	92	1	79	1	93	1	140
$Journalist1	2	111	2465	5	56	3	55	2	60	2	69	2	96
$Journalist2	3	99	1724	7	47	4	53	5	47	6	50	6	60
$Public_figure1	4	95	2169	6	54	5	49	10	40	5	51	4	77
$Politician1	5	92	1609	32	29	39	28	34	28	20	37	3	80
$Anonymous1	6	83	2228	4	58	2	56	7	44	12	46	5	62
$Public_figure2	7	79	1715	34	28	7	46	23	31	7	48	10	56
$Politician2	8	76	1403	31	29	10	39	4	47	4	52	27	43
$Public_figure3	9	75	2273	30	30	25	33	25	31	21	36	13	55
$Public_figure4	10	75	1998	28	30	12	39	8	42	3	56	14	53
**Left-to-Right**:													
@vladaRS	14	72	2287	446	9	586	8	489	8	52	26	9	57
@ukclj	32	59	2170	/	/	545	8	254	11	61	24	34	41
**Left-wing**:													
$PM-sep18-mar20	97	41	889	203	13	443	9	490	8	200	14	96	28
@necenzurirano_	103	40	876	/	/	/	/	/	/	225	13	48	37
$Public_figure5	131	37	1349	117	17	52	25	258	11	227	13	84	29
$Public_figure6	133	37	1014	1550	4	1480	4	793	6	365	10	68	32
@strankalevica	140	36	886	170	14	160	16	219	12	114	19	105	27
$Journalist3	141	35	1060	274	11	432	9	348	9	563	8	95	28
$Politician3	149	35	613	3621	1	4595	1	3869	1	267	12	83	29
$Journalist4	182	32	1236	239	12	144	17	218	12	157	16	124	25
@STA_novice	186	32	2204	367	10	255	13	325	10	143	17	162	23
@SpletnaMladina	246	28	1424	135	16	200	14	166	14	177	15	197	21

There is a large difference between the members of the Right-wing and Left-wing super-communities. The Right-wing members consistently take the top h-index ranks, while the Left-wing members barely make it into the top 100 h-index ranks. This reaffirms the super-community influence results from the subsection Identification of super-communities, and is consistent with our previous results. In the case of the European Parliament, higher Twitter activity was observed for the right-wing parties [18]. In the case of Brexit, the Leave proponents showed much higher activity and influence on Twitter than the Remain proponents [19].

As per PLoS ONE policy, individual Twitter users have to be anonymized. Therefore we replace each individual Twitter handle @User with an anonymous handle $Type. The user types for the top 890 users were determined manually [29]. There are three types of individual users: Politician, Public_figure, and Journalist, and an additional Anonymous type for users that cannot be easily identified. An artificial handle for the current and former prime ministers, $PM-*, was already introduced. Institutional Twitter accounts remain unchanged.

The Right-wing and Left-wing super-communities are led by the current ($PM-mar20-now) and former ($PM-sep18-mar20) prime minister of Slovenia, respectively. The other top members are either politicians, journalists, or public figures active on Twitter. In the Left-wing, there are some media account (@necenzurirano_, @STA_novice, @SpletnaMladina), and a political party account (@strankalevica—‘The Left’).

The only two users in Table 1 which are clearly unrelated to politics are @ukclj (University Medical Centre Ljubljana) and $Public_figure6 (a biochemist from the National Institute of Chemistry). They reached the rank of top 100 influencers only after the emergence of the Covid-19 pandemic (@ukclj at *t* = 91, and $Public_figure6 at *t* = 132). They post tweets about the medical issues, drugs and vaccines related to the pandemic. There are other influential users, tracking and commenting on the pandemic development, which emerged recently, but they did not yet make it into the overall top ten h-index list.

## Conclusions

Social media, and Twitter in particular, are a rich source of data that reflects social relations between the users. In the paper we exploit a specific type of networks where retweets are used as links between the users. We demonstrate that in the retweet networks meaningful communities are formed. We show that retweet influence reveals important differences between different communities as well as between individual Twitter users. The main focus of the paper is on the evolution of communities and influence through time, and we address several issues relevant for the field of dynamic networks.

One problem is the instability of detected static communities by a standard community detection Louvain algorithm. We propose to run an ensemble of Louvain trials, and detect stable communities through frequent co-occurrence of nodes across the trials. Preliminary evaluations of the Ensemble Louvain algorithm on some benchmark networks with known “ground truth” communities show promising results, and this is certainly one of the directions that needs to be further explored in the future.

We study network evolution by taking several static network snapshots with a sliding window. One has to decide on the window size and the temporal resolution between the snapshots. We decided on the 24 weeks window size and an exponential edge weight decay, with half-time of 4 weeks. The edge decay removes the effect of the trailing end of the window, and thus makes the choice of the window size less relevant. The choice of the half-time decay, on the other hand, is subject to experimentation, and depends on the volume of Twitter data. The chosen sliding window of one week provides high temporal resolution, but again the choice of this parameter is not crucial. We propose a temporal zoom-out to a lower time resolution, by computationally efficient selection of more distant timepoints where the network partitions exhibit larger differences. An analysis of how robust is this selection and what are meaningful ranges of distant timepoints is required in the future.

We apply and extend a measure of community similarity BCubed, which was originally introduced to evaluate quality of document clustering, but does not appear to be used in the field of complex networks. The *F*_1_ score can measure differences between network communities with only partially overlapping set of nodes. This is essential for comparing retweet networks, where new nodes keep appearing and disappearing from the network snapshots. An additional nice property of *F*_1_ is that it degenerates into a well-known set comparison coefficient, directly related to the Jaccard index.

A specially interesting result of this research is clear identification of super-communities from external influence links between the detected communities. The exiting problem, worth addressing in the future, is how to design a multi-stage super-community detection algorithm. This seems relevant for retweet networks in particular, where a standard community detection algorithm produces a large set of fractured communities.

There are two follow-up directions of the current research, already undertaken: classification of tweets by the level of hate speech and detection of discussion topics [30], and attribution of the hate speech to the detected communities and types of users [29]. The results show that most of the hate speech has the form of offensive tweets, and that over 60% of them can be attributed to a single right-leaning community of moderate size.

We illustrate our approach on a well-defined set of Slovenian tweets, of reasonable size, but not extremely large. Our next step is to apply the same approach on two different, but somehow related sets of Croatian and Serbian tweets. This will reveal which parameters need to be tuned to specific datasets, and what seem to be domain-invariant properties and methods, applicable to a wide range of domains.

## Methods

### Data collection

The three years of comprehensive Slovenian Twitter data cover the period from January 1, 2018 until December 28, 2020. In total, 12,961,136 tweets were collected. We used the TweetCat tool [35] for Twitter data acquisition.

The TweetCat tool is specialized on harvesting Twitter data of less frequent languages. It searches continuously for new users that post tweets in the language of interest by querying the Twitter Search API for the most frequent and unique words in that language. Once a set of new potential users posting in the language of interest are identified, their full timeline is retrieved and the language identification is run over their timeline. If it is evident that specific users post predominantly in the language of interest, they are added to the user list and their posts are being collected for the remainder of the collection period. In the case of Slovenian Twitter, the collection procedure started at the end of 2017 and is still running. As a consequence, we are confident that the full Slovenian tweetosphere is well covered.

### Ensemble Louvain

The Ensemble Louvain algorithm addresses the problem of instability of the Louvain community detection algorithm. The instability is manifested by different results of community detection in the same network, run with different initial seeds. This is due to theoretical issues with modularity maximization, and to heuristic nature of an efficient implementation of the algorithm.

We address this instability problem with a new approach called Ensemble Louvain. The steps of the algorithm are as follows:

1. run several trials of Louvain on the same network,
2. built a new network where a pair of the original nodes is linked if their total co-membership across all the Louvain trials is above a given threshold (e.g., 90%),
3. identify the disjoints sets which represent the resulting communities.

**Fig 7:**
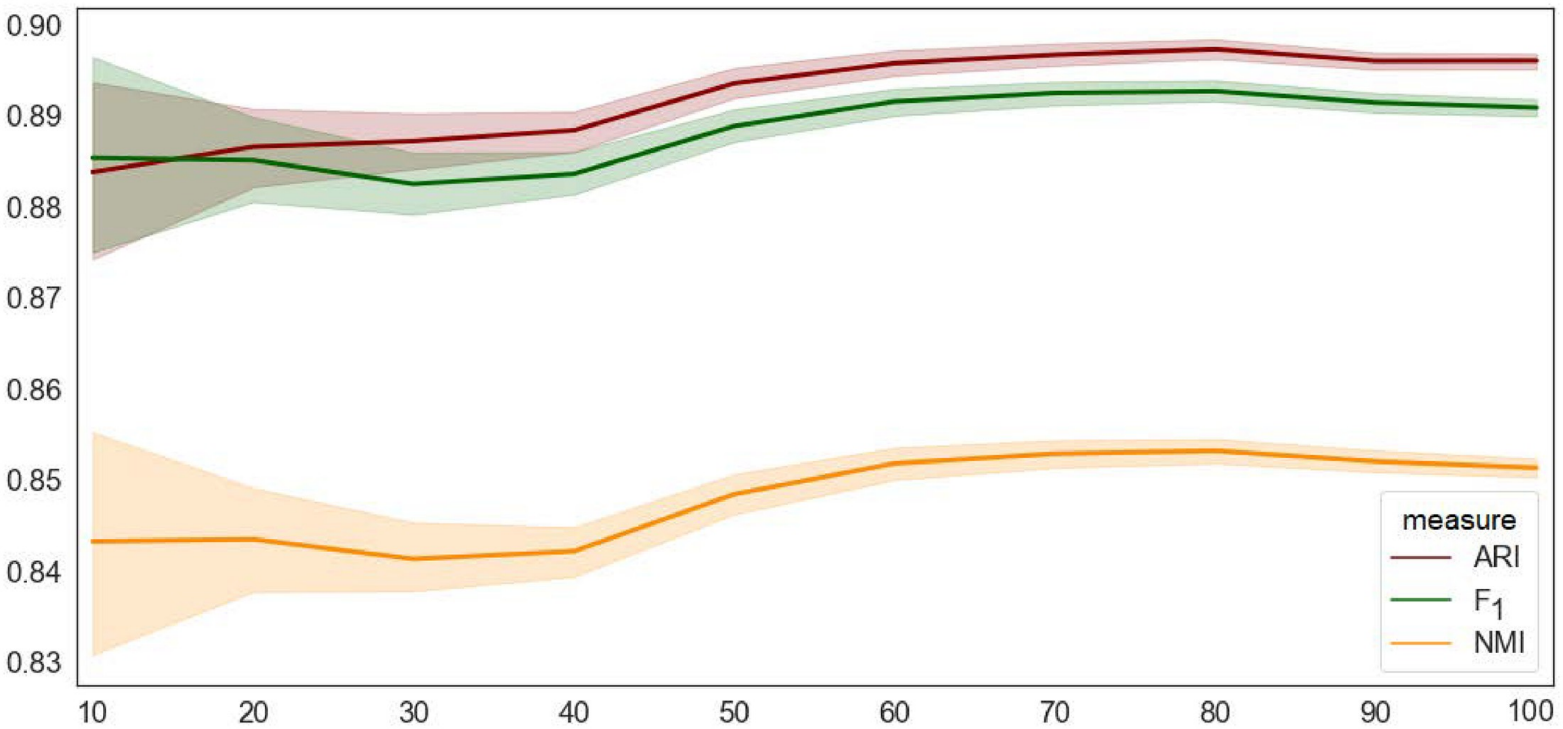
A comparison of the BCubed *F*_1_ measure with Adjusted Rand Index (ARI) and Normalized Mutual Information (NMI). The comparison is run on the initial *G*_0_ Slovenian retweet network. On the x-axis is the number of standard Louvain trials, *N* = 10, 20, …, 100. For each *N*, all the resulting partitions are pairwise compared by the three measures, ARI, *F*_1_, and NMI (y-axis). Solid lines show the mean values and shaded areas the 95% confidence intervals.

More trials eventually lead to more stable partitioning (see Fig 7), but increase the computation time. We found a reasonable trade-off between 50 and 500 trials, depending on the network size.

We are not the first to use ensembles for community detection. A combination of several different algorithms to create a refined partitioning was proposed in [36]. Re-sampling methods with variations of the same network were used by [37]. [38] create weighted consensus graphs and then detect communities in the consensus graph.

We measure the stability of Ensemble Louvain by Normalized Mutual Information (NMI) and Adjusted Rand Index (ARI). An initial comparison between the standard Louvain versus Ensemble Louvain is performed on three well-known datasets: the Football network (115 nodes), the Email EU core (1005 nodes), and a Slovenian retweet network (3992 nodes). 100 separate experiment runs show that Ensemble Louvain yields significantly more stable results, especially on the larger networks, where the variation between possible solutions grows.

We measure the performance with respect to the “ground truth” for the Football and Email EU Core networks. The initial results (presented by the mean ± standard deviation of the scores) show a significant improvement of Ensemble Louvain over the standard Louvain:

- The Football network,
  standard Louvain: NMI = 0.88±0.015 and ARI = 0.78±0.041,
  Ensemble Louvain: NMI = 0.92±0.008 and ARI = 0.89±0.019.
- The Email EU Core network,
  standard Louvain: NMI = 0.58±0.016 and ARI = 0.32±0.032,
  Ensemble Louvain: NMI = 0.72±0.005 and ARI = 0.52±0.012.

### BCubed measure of community similarity

The BCubed measure was originally proposed to evaluate effectiveness of document clustering [39]. Its properties were compared to a wide range of other extrinsic clustering evaluation metrics, with the conclusion that BCubed satisfies all the required qualitative properties [27]. Since data clustering and community detection in networks produce analogous results, one can also apply the BCubed measure to evaluate the detected communities. Communities can be evaluated against the “ground truth” when available, or compared to each other, as is the case with evolving communities.

The BCubed measure is applicable to individual nodes, communities, and network partitions in general. It decomposes the evaluation into calculating the precision and recall associated with each node in the network. The precision (*Pre*) and recall (*Rec*) are then combined into the *F*_1_ score:

$$\begin{array}{c} F_{1}=2\;\frac{Pre\cdot Rec}{Pre+Rec}. \end{array}$$

The *F*_1_ score is a special case of Van Rijsbergen’s effectiveness measure [40], where precision and recall can be combined with different weights. In the following we focus on definitions of precision and recall for different cases, and assume a balanced definition of the *F*_1_ score as the harmonic mean. We first define the BCubed measure for a node, and then proceed with definitions of *core-F*_1_, *standard F*_1_, and theoretical *max-F*_1_.

Let *L*(*n*) denote the “ground truth” community and *C*(*n*) the detected community of the node *n*, *n* ∈ *L*(*n*), *C*(*n*). *Pre* and *Rec* for a node are defined as follows:

$$\begin{array}{c} Pre\left(n\right)=\frac{\left|L(n)\cap C(n)\right|}{\left|C(n)\right|}, \end{array}$$

$$\begin{array}{c} Rec\left(n\right)=\frac{\left|L(n)\cap C(n)\right|}{\left|L(n)\right|}. \end{array}$$

#### *Core-F*_1_

Let first assume a special case when a pair of network partitions consist of the same set of nodes. In this case, we name the BCubed measure *core-F*_1_. Let *Ls* = {*L*_*i*_} denote a set of “ground truth” communities *L*_*i*_, and *Cs* = {*C*_*i*_} a set of detected communities *C*_*i*_. Constituent *Pre* and *Rec* for the partition *Cs* with respect to *Ls* are defined as:

$$\begin{array}{c} Pre\left(Cs|Ls\right)=\frac{1}{\left|Cs\right|}\sum_{n\in C_{i},C_{i}\in Cs}Pre\left(n\right), \end{array}$$

$$\begin{array}{c} Rec\left(Cs|Ls\right)=\frac{1}{\left|Ls\right|}\sum_{n\in L_{i},L_{i}\in Ls}Rec\left(n\right). \end{array}$$

The *F*_1_ measure proposed by Rossetti [28] is a special case of the *core-F*_1_. In our case, the *Pre* and *Rec* are computed with respect to all the communities *Ci* and *Li*, while Rossetti computes the *Pre* and *Rec* just between a pair of communities with the largest overlap.

#### *Standard F*_1_

In general, a pair of partitions *P*_0_, *P*_1_ has some overlapping nodes, and some nodes that are present in only one of the partitions. Let *Ls*, *Cs* denote communities with overlapping nodes, and *R*_0_, *R*_1_ the nodes specific to the respective partitions *P*_0_, *P*_1_. We have:

$$\begin{array}{c} P_{0}=Ls\cup R_{0},\;\;\;P_{1}=Cs\cup R_{1}. \end{array}$$

*Pre* and *Rec* of partition *P*_1_ with respect to the “ground truth” partition *P*_0_ are then computed as follows:

$$\begin{array}{c} Pre\left(Cs|P_{0}\right)=Pre\left(Cs|Ls\right), \end{array}$$

$$\begin{array}{c} Pre\left(P_{1}|P_{0}\right)=\frac{\left|Cs\right|}{\left|Cs|+\right|R_{1}|}Pre\left(Cs|Ls\right), \end{array}$$

$$\begin{array}{c} Rec\left(Cs|P_{0}\right)=\frac{\left|Ls\right|}{\left|Ls|+\right|R_{0}|}Rec\left(Cs|Ls\right), \end{array}$$

$$\begin{array}{c} Rec\left(P_{1}|P_{0}\right)=Rec\left(Cs|P_{0}\right). \end{array}$$

#### *Max-F*_1_

A theoretical maximum value of *F*_1_ can be computed under the assumption that all the overlapping nodes of the two partitions *P*_0_, *P*_1_ form one community. Let *C* = *L* denote the community with the intersecting nodes, *R*_0_ extra nodes in *P*_0_ (w.r.t. *P*_1_), and *R*_1_ extra nodes in *P*_1_ (w.r.t. *P*_0_):

$$\begin{array}{c} C=L=P_{1}\cap P_{0},\;\;\;P_{0}=L\cup R_{0},\;\;\;P_{1}=C\cup R_{1}. \end{array}$$

*Pre* and *Rec* of *P*_1_ with respect to the “ground truth” *P*_0_ are computed as:

$$\begin{array}{c} Pre\left(P_{1}|P_{0}\right)=\frac{\left|C\right|}{\left|C|+\right|R_{1}|}=\frac{\left|C\right|}{|P_{1}|}, \end{array}$$

$$\begin{array}{c} Rec\left(P_{1}|P_{0}\right)=\frac{\left|C\right|}{\left|L|+\right|R_{0}|}=\frac{\left|C\right|}{|P_{0}|}. \end{array}$$

The *max-F*_1_ score is then:

$$\begin{array}{c} F_{1}\left(P_{1}|P_{0}\right)=2\;\frac{Pre\left(P_{1}|P_{0}\right)\cdot Rec\left(P_{1}|P_{0}\right)}{Pre\left(P_{1}|P_{0}\right)+Rec\left(P_{1}|P_{0}\right)}=2\;\frac{\left|C\right|}{|P_{1}\left|+\right|P_{0}|}=2\;\frac{|P_{1}\cap P_{0}|}{|P_{1}\left|+\right|P_{0}|}. \end{array}$$

This measure of similarity of two sets, *P*_0_ and *P*_1_, is also known as Sørensen-Dice coefficient [41, 42]. It is directly related to the Jaccard index:

$$\begin{array}{c} Jacc\left(P_{1}|P_{0}\right)=\frac{|P_{1}\cap P_{0}|}{|P_{1}\cup P_{0}|}. \end{array}$$

The transformation between the Jaccard index and *F*_1_ is as follows:

$$\begin{array}{c} Jacc=\frac{F_{1}}{2-F_{1}},\;\;\;F_{1}=\frac{2\cdot Jacc}{1+Jacc}. \end{array}$$

The BCubed-based *F*_1_ measure therefore has two special cases, *core-F*_1_ for comparing completely overlapping network partitions, and *max-F*_1_ for comparing two partitions with emerging (new) and disappearing (lost) nodes. The later case is specially relevant in evolving retweet networks, when new users appear and some users leave the network at different time windows.

The *F*_1_ can be compared to standard community evaluation measures, such as Adjusted Rand Index (ARI) and Normalized Mutual Information (NMI). In Fig 7 we compare the three measures on the same network, running several trials of the standard Louvain with different initial seeds. The *F*_1_ in this case is actually the *core-F*_1_, compatible to ARI and NMI. ARI and NMI cannot be applied in the case when network partitions differ in the sets of respective nodes.
